# Would you like to be a general practitioner? Baseline findings of a longitudinal survey among Danish medical trainees

**DOI:** 10.1186/s12909-024-05074-1

**Published:** 2024-02-05

**Authors:** Sofie Gjessing, Trine Lignell Guldberg, Torsten Risør, Regitze Gyldenholm Skals, Jette Kolding Kristensen

**Affiliations:** 1 Center for General Practice, Aalborg University, Aalborg, Denmark; 2 Department of Postgraduate Medical Education, Aalborg University Hospital, Aalborg, Denmark; 3 Section for General Practice & Research Unit for General Practice, Department of Public Health, University of Copenhagen, Copenhagen, Denmark; 4 Section for General Practice, Department of Community Medicine, UiT, The Arctic University of Norway, Tromsø, Norway; 5 Department of Clinical Biostatistics, Aalborg University Hospital, Aalborg, Denmark

**Keywords:** Medical specialty choice, General practice, Medical education, Specialty choice process, Career choice

## Abstract

**Background:**

Recruiting and securing primary care physician workforce has been the center of international attention for decades. In Denmark, the number of general practitioners has decreased by 8.5% since 2013. However, a rising population age and increasing prevalence of chronic diseases and multimorbidity place an even greater future need for general practitioners in Denmark. The choice of general practice as specialty has been associated with a range of both intrinsic and extrinsic factors, however, few studies have examined the recruitment potential that lies within medical trainees’ who are undecided about general practice specialization. The aim of this study was, therefore, to explore how medical trainees who are undecided about general practice specialization (GP-positive/undecided) differ from medical trainees who are either committed (GP-committed) or not committed to a general practice career (GP-non-committed) regarding factors related to future work life.

**Methods:**

The present study concerns baseline findings from a longitudinal survey study. An online questionnaire was e-mailed to a national cohort of medical trainees during their transition from under- to postgraduate education. The associations between orientations towards general practice specialization and work-related factors and potential influencing factors, respectively, were analyzed using uni- and multivariable modified Poisson regression models.

**Results:**

Of 1,188 invited participants, 461 filled out key study variables concerning specialty preferences and rejections, corresponding to a response rate of 38.8%. We found significant positive associations between GP-positive/undecided orientation and valuing a good work/life balance and the opportunity to organize own working hours when compared to GP-non-committed respondents. Compared to the GP-committed orientations, the GP-positive/undecided orientation was associated with a positive attitude towards technology, working shift hours, and an openness towards several career paths. Across all orientations, undergraduate exposure to the specialties was found to be highly influential on the specialty preferences.

**Conclusion:**

GP-positive/undecided medical trainees value autonomy over their working hours more than the GP-non-committed, but less than the GP-committed. However, the GP-positive/undecided respondents present more openness to different career opportunities and the use of technology in daily work. We suggest using this knowledge in the planning of recruitment strategies aiming to increase interest in general practice specialization.

## Background

Population aging and the increasing prevalence of chronic diseases and multimorbidity place greater resource demands on the healthcare systems [1, 2]. This leads to a strengthened need for a healthcare workforce including primary care physicians. A strong primary care, manned by general practitioners (GPs) is associated with better population health in the United States and England, while a deficit in GPs is associated with an increase in hospital utilization [3–5]. In Denmark, as in most other European countries, Australia, and the United States, GPs are specialists in general practice/family medicine working in a primary care setting [6–8]. They are self-employed physicians who manage their own practice, either alone (40%) or in a partnership (60%). As a GP in Denmark, you have a contract with the health authorities and will be remunerated through the national health system. There are restrictions where to locate the clinic, but the organization of the clinic, including daily work, is up the GP to decide [6]. Specialists in general practice/family medicine can, however, also be employed at for instance hospitals, in municipalities, or private companies, as was the case for 17.5% of the Danish general practice specialists in 2017 [9, 10]. In 2022, the number of GPs in Denmark was 3,284 after an 8.5% decline over the previous decade (2013–2022) [11]. However, to meet the future demands on the primary healthcare system, a need for a total of 5,000 GPs in 2030 has been estimated [12].

Much attention has been paid to this issue of recruiting and securing the primary care physician workforce [13, 14], and research on primary care career choice is extensive. The factors related to primary career choice are multiple [15], and undergraduate education, especially, has been found to play a pivotal role in influencing primary care career choice [16]. Yet, the process of choosing a medical specialty is dynamic with career intentions being subject to change during undergraduate medical education [17]. Also, a recent longitudinal study on the dynamics of career intentions during medical school found that career intentions become more stable in the final clinical years [18].

### Conceptual framework

In 2010, Bennett and Phillips presented a conceptual model of the process of primary care specialty choice [13]. The authors found that medical students had different predispositions to primary care career choice and that they, in turn, were influenced by different factors. Overall, the conceptual model categorizes medical students into four categories: primary care committed, primary care positive, undecided, and non-primary care committed students. When time is included in the model, the authors suggest that medical students predisposed to primary care are likely to choose a career in primary care. Likewise, medical students inclined towards non-primary care are prone to choose a non-primary care career. Thus, according to the model, the greatest immediate recruitment potential is within the group of medical students who are either positive towards or undecided about a future job within primary care, since they can be influenced to either a primary care or non-primary care career path. The understanding that medical students’ decisions depend on their initial interest in primary care and that a longitudinal interaction between the medical student and environment takes place during medical education has subsequently been included in the expanded conceptual framework of medical students’ primary care career choice by Pfarrwaller and colleagues [19]. This framework offers the most comprehensive understanding of the specialty choice process to date suggesting that it is a continuous match between the students’ personal interests and perception of a specialty’s characteristics. According to the model, this is subject to multiple interacting influences both within and outside medical school.

Even though Bennett and Phillips already in 2010 suggested future research to be formed on the conceptual basis of a theoretical model, few studies have subsequently examined the recruitment potential of the group of undecided medical students [18, 20, 21]. Therefore, knowledge is needed of the group of undecided medical trainees and their priorities regarding their future work life to examine the potential to recruit more specialists to general practice.

### Aim

With the present study, we use data from a longitudinal study to investigate the primary care physician recruitment potential suggested in the theoretical models of the specialty choice process. Thus, we aimed to explore how medical trainees who are undecided about general practice specialization differ from medical trainees who are committed or non-committed to a general practice career. Using the lens of the conceptual framework to investigate factors associated with these specialty orientations may provide knowledge useful in developing tailored career advice and recruitment strategies promoting general practice careers.

## Methods

This study forms part of a longitudinal cohort study based on a questionnaire developed to measure specialty orientation and associated factors over time. While the present paper concerns baseline findings, the development of the questionnaire and data collection are described in detail in an earlier paper by Gjessing et al. [22].

### Educational context

In Denmark, undergraduate medical education is conducted at four medical schools. Each medical school has its own six-year curriculum. Medical students can be employed at hospitals or in general practice during medical school either to fulfill the duties of a physician full or part-time (locum work) or to do other tasks (students’ job). After graduation, medical trainees begin basic clinical training consisting of six months of employment at a hospital department and six months of employment in general practice. This is a prerequisite for starting the general practice postgraduate training program which includes a half-year introductory training program and a 4.5-year main training program [23]. Medical trainees are free to choose any specialty after graduation and to enter and complete several introductory training programs before starting a main training program. There is no time limit for becoming a medical specialist and changing specialty during postgraduate education is possible. Each year, 350 main training positions in general practice are offered, and in 2022, 73% of them were filled [24].

### Participants and setting

All medical trainees beginning basic clinical training in 2022 were recruited into a cohort study and invited to complete the electronic survey before starting the basic clinical training program with follow-up after approximately 15 months. From a total of 1,188 medical trainees, 461 enrolled in the study (38.8%). For the present study, we consider cross-sectional data collected at baseline in November/December 2021 and May/June 2022.

### Outcome variables

The orientation toward a general practice career was identified by asking participants to name their first, second, and third priorities for specialization as well as the specialties they with certainty would rule out for specialization. The orientations were categorized into GP-committed (general practice as specialty preference), GP-positive/undecided (general practice not mentioned as first preference nor excluded), and GP-non-committed (general practice excluded from specialty considerations). To address the purpose of the study, we defined two dichotomous outcome variables for statistical analyses. The first outcome was defined as GP-positive/undecided compared to GP-non-committed (outcome model 1). Likewise, the second outcome was GP-positive/undecided compared to GP-committed (outcome model 2).

### Independent variables

The present section concerns the items examining the factors that in interviews and existing literature were found to have the potential to be associated with general practice career choice over time. The included factors covered the following topics: background, work content, working hours, patient interaction, professional relationships, and career opportunities. A 5-point Likert response format was used to assess the participants’ attitudes towards the statements (i.e., “I like to follow procedures and guidelines” from 1 = ‘strongly disagree’ to 5 = ‘strongly agree’). The participants were asked to assess the importance of the four Likert items regarding a future career (work/life-balance, alternative career opportunities, self-employment, and geography) by stating to what degree the lack of the stated attribute would make them exclude the specialty from future career plans.

The participants were also presented with 14 factors and asked to rate the degree to which it had influenced their stated specialty preference (i.e., “The meeting with clinicians/teachers from the specialty” from 1 = ‘not at all’ to 5 = ‘to a great extent’).

### Statistical analyses

**Fig. 1:**
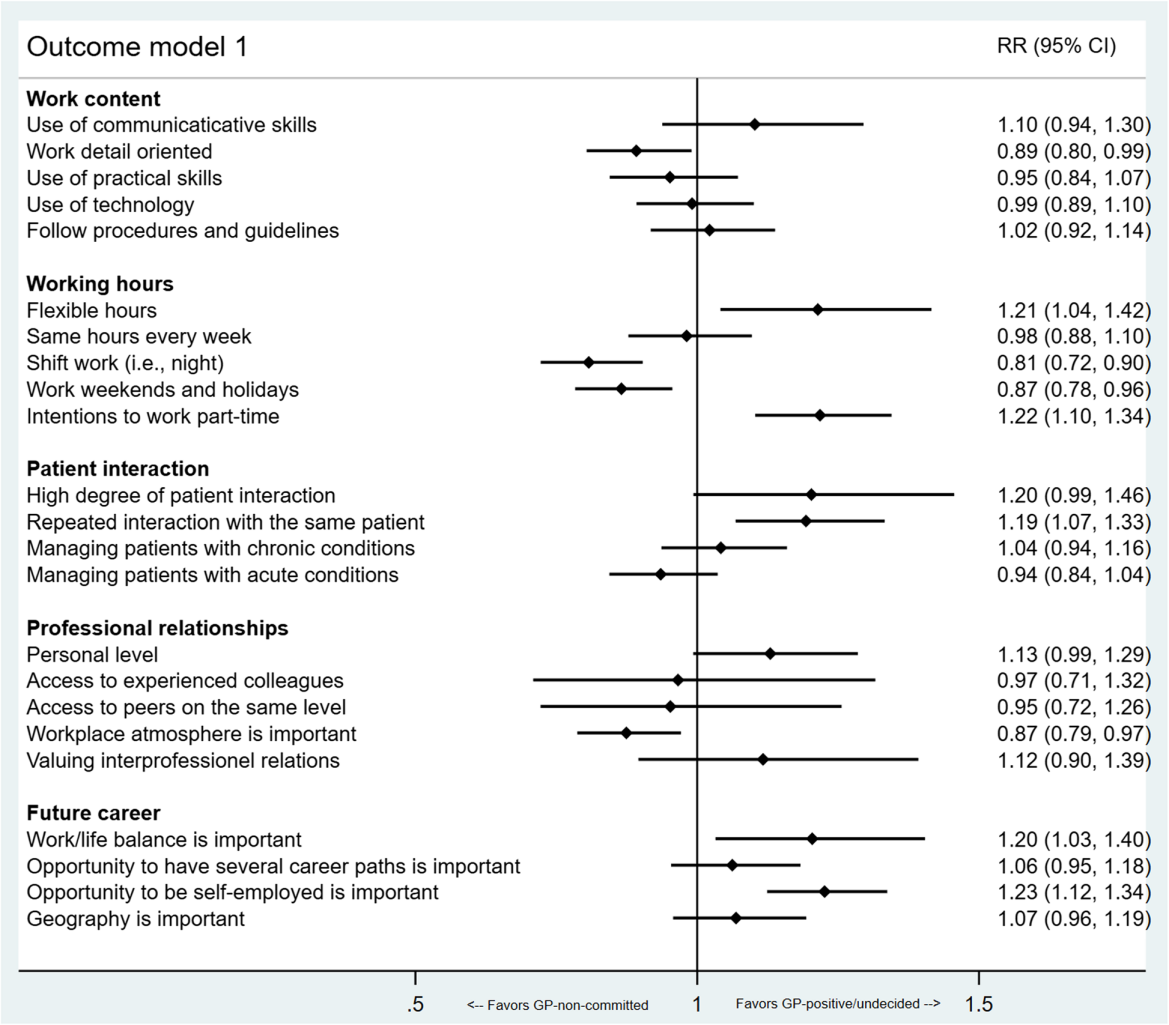
Outcome model 1. Multivariable modified Poisson regression model comparing GP-positive/undecided orientation with GP-non-committed. The model is adjusted for gender and graduation university, and the figure shows the estimated relative risks with 95% confidence intervals

**Fig. 2:**
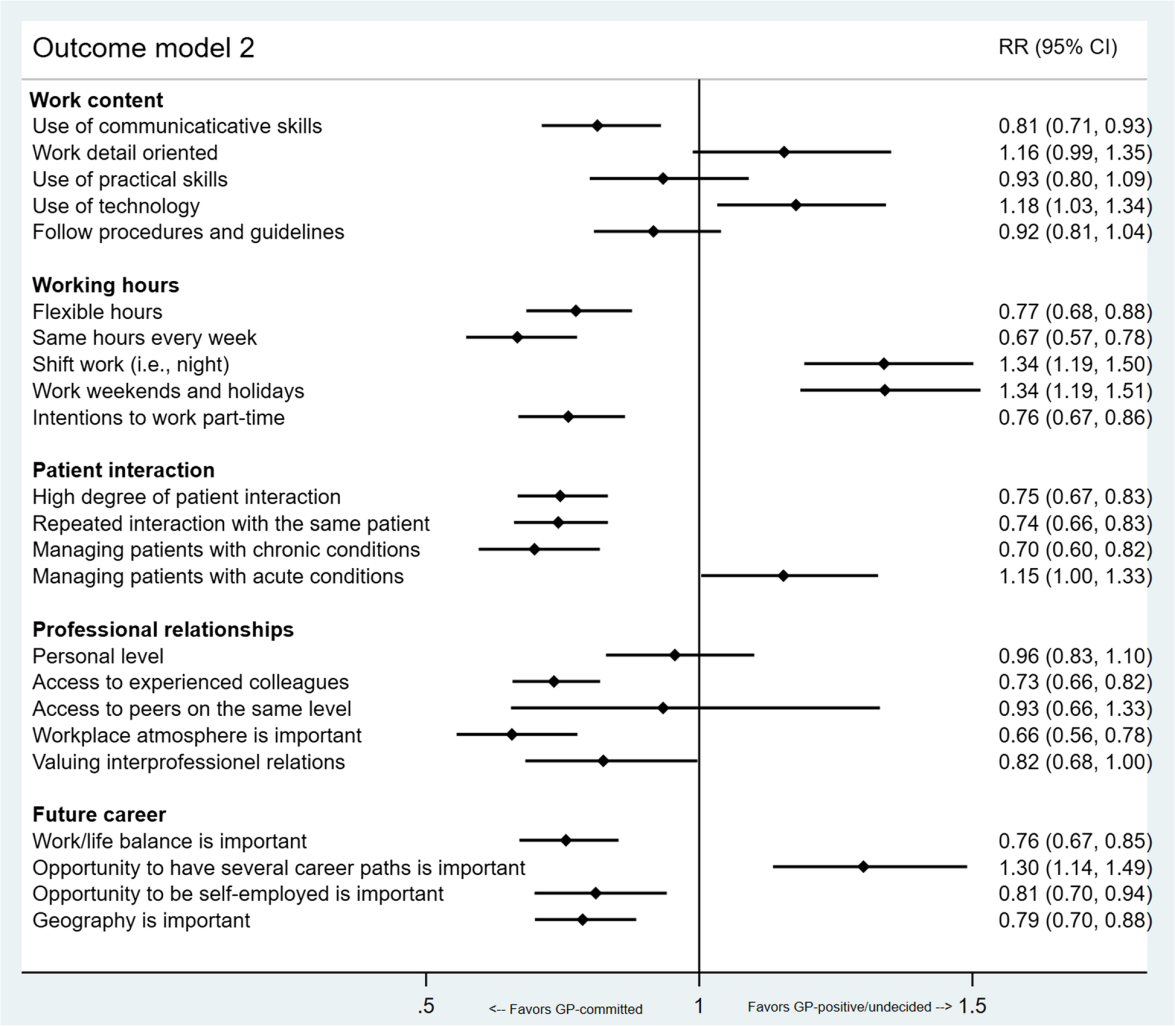
Outcome model 2. Multivariable modified Poisson regression model 2 comparing GP-positive/undecided orientation with GP-committed. The model is adjusted for gender and graduation university, and the figure shows the estimated relative risks with 95% confidence intervals

Participant characteristics were reported by counts and proportions and compared across specialty orientation groups using a Chi-square test. Age was reported by mean, standard deviation, and range. Histograms and Q-Q plots were used to graphically test whether data on age were normally distributed within the groups, and analysis of variance (ANOVA) was used to compare mean ages. The distribution of the Likert items was presented by means (SD) and medians (IQR) and categorized into dichotomous variables (‘strongly agree’ and ‘agree’ or ‘to a great extent’ and ‘to some extent’ = 1, otherwise = 0). This was chosen to avoid violation of the model assumptions of the modified Poisson regression. The categorical version was entered as the independent variable in each model. Gender and graduation university were included as covariates due to their possible association with specialty choice reported in the literature [25, 26]. The association between general practice orientation and the associated and influencing factors was examined using uni- and multivariable modified Poisson regression models due to its ability to estimate relative risks on common outcome [27, 28]. An estimated relative risk greater than one indicates that respondents who ‘agreed’ or ‘strongly agreed’ with the given statements have a higher probability of being positive towards or undecided about a general practice specialization (favors GP-positive/undecided). On the contrary, if the ratio instead is less than one, there is a higher probability of the respondents not being GP-positive/undecided. Thus, a ratio less than one favors either the GP-non-committed (Fig. 1) or GP-committed (Fig. 2) specialty orientation. All analyses were carried out using version 17 of STATA [29]. In all analyses, a two-sided p-value below 0.05 was considered statistically significant.

## Results

### Participant characteristics

Of 1,188 invited medical trainees, 477 responded to the survey invitation, and 461 had filled out the key study variables concerning their specialty orientation yielding a response rate of 38.8%. Flowchart of participation and non-response analyses are provided in a former paper [22]. Table 1 presents the demographic characteristics of all participants, and the characteristics of the respondents with respect to their specialty orientations. About 25% of the participants had general practice as specialty preference (GP-committed), while 61.2% were either positive about general practice specialization or undecided (GP-positive/undecided). In total, 13.9% had excluded general practice from specialization considerations (GP-non-committed).

**Table 1:** Baseline characteristics of participants by specialty orientation

		**GP-committed**		**GP-positive/undecided**		**GP-non-committed**		**Total**		*p*-value^*^
		n	%	n	%	n	%	n	%	
		115	24.9	282	61.2	64	13.9	461	100	
Gender	Female	88	76.5	183	65.1	43	67.2	314	68.1	0.085
	Male	27	23.5	98	34.9	21	32.8	146	31.7	
Nationality	Danish	110	95.7	264	93.6	59	92.2	433	93.9	0.611
	Other	5	4.4	18	6.4	5	7.8	28	6.1	
In which type of area did you grow up ?	Urban	77	67.0	193	69.2	52	81.3	322	69.8	0.108
	Rural	38	33.0	86	30.8	12	18.8	136	29.5	
Which language was spoken in your home when growing up?	Danish	96	83.5	116	80.1	51	79.7	373	80.9	**0.006**
	Other/multiple	19	16.5	56	19.9	13	20.3	88	19.1	
Is any of your parents a physician?	Yes	7	6.1	40	14.2	16	25.0	63	13.7	**0.002**
	No	108	93.9	242	85.8	48	75.0	398	86.3	
Civil status	Single	14	12.2	70	24.8	18	28.1	102	22.1	**0.010**
	In a relationship	101	87.8	212	75.2	46	71.9	359	77.9	
Children	No	85	73.9	222	78.7	50	78.1	357	77.4	0.576
	Yes (including expecting first child)	30	26.1	60	21.3	14	21.9	104	22.6	
Do you own your house?	Yes	33	28.7	74	26.4	13	20.3	120	26.0	0.466
	No	82	71.3	206	73.6	51	79.7	339	73.5	
Age in years	Range	24–39		24–47		24–37		24–47		0.823^a^
	Median (IQR)	27 (2)		27 (2)		27 (2)		27 (2)		
	Mean (SD)	27.4 (2.39)		27.4 (2.58)		27.3 (1.90)		27.4 (2.44)		

^*^Chi-square test used unless otherwise noted

^a^One-way ANOVA used to compare mean ages

### Factors associated with GP-positive/undecided orientation

Table 2 depicts participants’ attitudes towards factors concerning their future work-life displayed on specialty orientations. Overall, professional relationships were very important to all participants regardless of specialty orientation, and likewise, all participants wanted their future work to be interesting.

**Table 2:** Distribution of participants' attitudes towards factors related to future work life by specialty orientations. The table provides the number and percentage of participants who agreed with each statement (i.e., 4 or 5 from 1-5). Mean and median response ratings are displayed with SD and IQR, respectively

Statements	**GP-committed (*n* = 115)**					**GP positive and undecided (*n* = 282)**					**GP-non-committed (*n* = 64)**					**Total (*n* = 461)**				
	No. of participants (%) agreeing with statement	Mean (SD)		Median (IQR)		No. of participants (%) agreeing with statement	Mean (SD)		Median (IQR)		No. of participants (%) agreeing with statement	Mean (SD)		Median (IQR)		No. of participants (%) agreeing with statement	Mean (SD)		Median (IQR)	
**Work content**																				
I like my daily work to be interesting	113/113 (100%)	4.8	(0.4)	5.0	(0.0)	279/279 (100.0%)	4.9	(0.4)	5.0	(0.0)	63/63 (100.0%)	4.9	(0.3)	5.0	(0.0)	455/455 (100%)	4.9	(0.4)	5.0	(0.0)
I like to use my communicative skills at work	106/113 (93.8)	4.6	(0.6)	5.0	(1.0)	238/279 (85.3%)	4.3	(0.7)	4.0	(1.0)	49/62 (94.2%)	4.4	(0.9)	5.0	(1.0)	393/454 (86.6%)	4.4	(0.8)	5.0	(1.0)
I like to be detail oriented in my work	73/113 (64.6%)	3.8	(0.8)	4.0	(1.0)	210/279 (75.3%)	4.0	(0.9)	4.0	(1.0)	54/63 (85.7%)	4.4	(0.8)	4.0	(2.0)	337/455 (74.1%)	4.0	(0.9)	4.0	(2.0)
I like to use my practical skills at work	93/113 (82.3%)	4.1	(0.7)	4.0	(1.0)	225/278 (80.9%)	4.1	(0.9)	4.0	(1.0)	54/63 (85.8%)	4.5	(0.9)	5.0	(1.0)	372/454 (81.9%)	4.2	(0.9)	4.0	(1.0)
I like to use technology in my daily work	39/113 (34.5%)	3.3	(1.0)	3.0	(1.0)	146/279 (52.3%)	3.6	(0.9)	4.0	(1.0)	33/63 (52.4%)	3.8	(1.0)	4.0	(2.0)	218/455 (47.9%)	3.6	(1.0)	3.0	(1.0)
I like to follow procedures and guidelines	80/113 (70.8%)	3.8	(0.7)	4.0	(1.0)	176/279 (63.1%)	3.7	(0.9)	4.0	(1.0)	39/63 (61.9%)	3.9	(0.9)	4.0	(2.0)	295/455 (64.8%)	3.8	(0.8)	4.0	(1.0)
**Working hours**																				
I would like to have the opportunity to have flexible work hours (e.g., organize my own work schedule)	105/113 (92.9%)	4.6	(0.7)	5.0	(1.0)	223/277 (80.5%)	4.2	(0.8)	4.0	(1.0)	39/62 (62.9%)	3.9	(0.9)	4.0	(2.0)	367/452 (81.2%)	4.2	(0.8)	4.0	(1.0)
It is important to me that my work schedule is more or less the same every week	70/113 (61.9%)	3.7	(1.0)	4.0	(1.0)	85/277 (30.7%)	3.0	(1.0)	3.0	(2.0)	20/61 (32.8%)	2.8	(1.2)	3.0	(2.0)	175/451 (38.8%)	3.2	(1.1)	3.0	(2.0)
It is an advantage for me to work shifting hours (e.g., night and weekends)	19/113 (16.8%)	2.5	(1.0)	3.0	(1.0)	107/277 (38.6%)	3.1	(1.1)	3.0	(2.0)	41/62 (66.1%)	3.7	(1.1)	4.0	(1.0)	167/452 (36.9%)	3.0	(1.1)	3.0	(2.0)
I don't mind working when others are not (e.g., during weekends and holidays)	28/113 (24.8%)	2.6	(1.2)	2.0	(1.5)	140/277 (50.5%)	3.3	(1.1)	4.0	(1.0)	43/62 (69.4%)	3.9	(1.0)	4.0	(2.0)	211/452 (46.7%)	3.2	(1.2)	3.0	(2.0)
I see myself work part-time at some point in the future	80/113 (70.8%)	3.9	(1.1)	4.0	(2.0)	127/277 (45.8%)	3.3	(1.3)	3.0	(2.0)	13/62 (21.0%)	2.5	(1.2)	2.0	(2.0)	220/452 (48.7%)	3.4	(1.3)	3.0	(2.0)
**Patient interaction**																				
I value a high degree of interaction with patients	111/113 (98.2%)	4.7	(0.5)	5.0	(1.0)	241/275 (87.6%)	4.4	(0.8)	5.0	(1.0)	47/62 (75.8%)	4.1	(1.0)	4.0	(1.3)	399/450 (88.7%)	4.4	(0.8)	5.0	(1.0)
I value seeing the same patients repeatedly	97/113 (85.8%)	4.3	(0.8)	4.0	(1.0)	162/275 (58.9%)	3.7	(0.9)	4.0	(1.0)	22/62 (35.5%)	3.2	(1.1)	3.0	(1.0)	281/450 (62.4%)	3.8	(0.9)	4.0	(2.0)
I value managing patients with chronical conditions	68/113 (60.2%)	3.6	(0.8)	4.0	(1.0)	81/275 (29.5%)	3.1	(1.0)	3.0	(2.0)	15/62 (24.2%)	2.7	(1.1)	3.0	(1.3)	164/450 (36.4%)	3.2	(1.0)	3.0	(1.0)
I value managing patients with acute conditions	62/113 (54.9%)	3.5	(1.0)	4.0	(1.0)	183/275 (66.5%)	3.8	(1.0)	4.0	(2.0)	46/62 (74.2%)	3.9	(1.1)	4.0	(2.0)	291/450 (64.7%)	3.7	(1.0)	4.0	(1.0)
**Professional relationships**																				
It is important to me that my colleagues are familiar with me on a personal level	89/113 (78.8%)	4.0	(0.8)	4.0	(0.5)	205/275 (74.5%)	3.9	(0.9)	4.0	(1.0)	38/61 (62.3%)	3.7	(0.9)	4.0	(1.0)	332/449 (73.9%)	3.9	(0.8)	4.0	(1.0)
It is important to me to have access to more experienced colleagues	113/113 (100%)	4.8	(0.4)	5.0	(0.0)	269/275 (97.8%)	4.8	(0.5)	5.0	(0.0)	60/61 (98.4%)	4.8	(0.4)	5.0	(0.0)	442/449 (98.4%)	4.8	(0.5)	5.0	(0.0)
It is important to me to have the opportunity to have professional discussions with peers	111/113 (98.2%)	4.7	(0.5)	5.0	(1.0)	268/275 (97.5%)	4.7	(0.6)	5.0	(0.0)	60/51 (98.4%)	4.8	(0.5)	5.0	(0.0)	439/449 (97.8%)	4.7	(0.6)	5.0	(0.5)
The atmosphere at the workplace is important to me	113/113 (100%)	5.0	(0.2)	5.0	(0.0)	273/275 (99.3%)	4.9	(0.4)	5.0	(0.0)	61/61 (100.0%)	4.9	(0.3)	5.0	(0.0)	447/449 (99.6%)	4.9	(0.3)	5.0	(0.0)
I like working together with other professions, for instance nurses	109/113 (96.5%)	4.7	(0.6)	5.0	(1.0)	254/275 (92.4%)	4.6	(0.7)	5.0	(1.0)	54/61 (88.5%)	4.5	(0.7)	5.0	(1.0)	417/449 (92.9%)	4.6	(0.6)	5.0	(1.0)
**Future career**																				
I would exclude a specialty from my considerations if the work did not harmonize with my personal life	107/113 (94.7%)	4.6	(0.7)	5.0	(1.0)	221/274 (80.7%)	4.1	(0.9)	4.0	(1.0)	39/61 (63.9%)	3.6	(1.1)	4.0	(1.0)	367/448 (81.9%)	4.2	(0.9)	4.0	(1.0)
I would exclude a specialty from my considerations if it did not provide several career opportunities (e.g., research, or teaching)	39/113 (34.5%)	3.2	(1.0)	3.0	(2.0)	164/274 (59.9%)	3.6	(1.0)	4.0	(1.0)	32/61 (52.5%)	3.4	(1.3)	4.0	(2.5)	235/448 (52.4%)	3.5	(1.0)	4.0	(1.0)
I would exclude a specialty from my considerations if it did not provide the opportunity to be self-employed in the future	48/113 (42.5%)	3.3	(1.0)	3.0	(1.0)	79/275 (28.7%)	2.8	(1.1)	3.0	(2.0)	5/61 (8.2%)	2.1	(1.1)	2.0	(2.0)	132/449 (29.4%)	2.8	(1.2)	3.0	(2.0)
I would exclude a specialty from my considerations if it was not compatible with my intended area of residence	92/113 (74.8%)	4.1	(0.8)	4.0	(1.0)	179/275 (65.1%)	3.7	(1.0)	4.0	(1.0)	35/61 (57.4%)	3.4	(1.2)	4.0	(2.0)	306/449 (68.2%)	3.7	(1.0)	4.0	(1.0)

What stood out was the groups’ different attitudes toward especially working hours, patient interaction and future career opportunities. The forest plots presented in Figs. 1 and 2 show the associations between these factors and outcome models 1 and 2, respectively. Looking at outcome model 1 in Fig. 1, we found that participants with interest in flexible working hours (e.g., organization of own working time), part-time work, a continuity-based doctor-patient relationship, a good work/life-balance and the opportunity be self-employed were likely to be positive/undecided about a general practice career rather than non-committed to such.

Noticeable, in outcome model 2 (Fig. 2), we found the same items to be significantly associated with being committed rather than positive/undecided GP-orientation. This association in favor of general practice commitment was also found among participants with positive attitudes towards the use of communicative skills, interest in a high degree of patient interaction, and access to experienced colleagues. However, participants with positive attitudes towards technology-use at work, shift work (also when others are off duty), or alternative career paths in the future participants were significantly more likely to be positive/undecided than committed to a general practice career.

### Influence on specialty preferences

Table 3 presents the degree to which a range of factors has influenced the participants’ specialty preferences. The meeting with role models from the specialties and exposure to the specialties through clerkships during medical school were found to be highly influential to all participants. Experience from unspecified student’s jobs other than locum work was found to be most influential to the GP-committed participants (81.1%), whereas knowledge about specialties obtained through participants’ research activities was only found to have influenced 30.9% of the GP-committed participants. In contrast, 58.0% and 71.2% of the GP-positive/undecided and GP-non-committed, respectively, stated that research activities had influenced their specialty preferences. Results also revealed that especially GP-committed participants expressed that exposure to the specialty as either a patient or relative had influenced their stated first preference for specialization.

**Table 3:** Factors influencing specialty preferences. The number and percentage of participants rating each item as influential (i.e., 4 or 5 from 1-5) are displayed by specialty orientations. Mean and median response ratings are provided with SD and IQR, respectively. Relative risks with 95% confidence intervals from multivariable modified Poisson regression models are shown with corresponding *p*-values

**Factors influencing the listed specialty preference**	**GP-committed (*n* = 115)**					**GP positive and undecided (*n* = 282)**					**GP-non-committed (*n* = 64)**					**Total (*n* = 461)**					Outcome model 1^a^		Outcome model 2^b^	
	No. (%) of participants rating factor as influential	Mean (SD)		Median (IQR)		No. (%) of participants rating factor as influential	Mean (SD)		Median (IQR)		No. (%) of participants rating factor as influential	Mean (SD)		Median (IQR)		No (%) of participants rating factor as influential	Mean (SD)		Median (IQR)		Relative risk (95% CI)	*p*-value	Relative risk (95% CI)	*p*-value
Rolemodels	101/113 (89.4%)	4.3	(0.8)	4.0	(1.0)	251/270 (93.0%)	4.4	(0.8)	5.0	(1.0)	55/60 (91.7%)	4.4	(1.0)	5.0	(1.0)	407/443 (91.9%)	4.3	(0.8)	4.0	(1.0)	1.04 (0.84;1.29)	0.701	1.16 (0.87;1.55)	0.312
Clerkships	100/113 (88.5%)	4.3	(0.8)	4.0	(1.0)	240/269 (89.2%)	4.3	(0.9)	5.0	(1.0)	52/59 (88.1%)	4.2	(1.1)	4.0	(1.0)	392/441 (88.9%)	4.3	(0.9)	5.0	(1.0)	1.02 (0.86;1.21)	0.808	1.02 (0.83;1.26)	0.836
Formal teaching	65/111 (58.6%)	3.5	(1.1)	4.0	(1.0)	179/270 (66.3%)	3.6	(1.1)	4.0	(1.0)	33/59 (55.9%)	3.4	(1.3)	4.0	(2.0)	277/440 (63.0%)	3.6	(1.1)	4.0	(1.0)	1.08 (0.96;1.21)	0.180	1.12 (0.98;1.29)	0.097
Student's job	77/95 (81.1%)	4.1	(1.2)	4.0	(1.0)	124/226 (54.9%)	3.3	(1.4)	4.0	(2.0)	25/43 (58.1%)	3.4	(1.5)	4.0	(3.0)	226/364 (62.1%)	3.5	(1.4)	4.0	(2.0)	0.99 (0.89;1.10)	0.802	0.74 (0.65;0.84)	**0.000**
Peers and colleagues	67/106 (63.2%)	3.5	(1.2)	4.0	(1.0)	124/257 (48.2%)	3.1	(1.3)	3.0	(2.0)	24/55 (43.6%)	2.9	(1.4)	3.0	(3.0)	215/418 (51.4%)	3.2	(1.3)	4.0	(2.0)	1.04 (0.94;1.15)	0.499	0.84 (0.74;0.96)	**0.009**
Research activities	29/94 (30.9%)	2.6	(1.3)	2.0	(3.0)	149/257 (58.0%)	3.4	(1.4)	4.0	(3.0)	37/52 (71.2%)	3.9	(1.3)	4.0	(2.0)	215/443 (48.5%)	3.3	(1.5)	4.0	(3.0)	0.97 (0.88;1.08)	0.631	1.36 (1.19;1.56)	**0.000**
Network (other than peers and colleagues)	42/100 (42.0%)	3.0	(1.4)	3.0	(2.0)	93/246 (37.8%)	2.7	(1.5)	3.0	(2.0)	18/52 (34.6%)	2.6	(1.5)	2.0	(3.0)	153/442 (34.6%)	2.8	(1.5)	3.0	(3.0)	1.03 (0.92;1.14)	0.626	0.92 (0.80;1.06)	0.246
Experiences as locum doctor	40/76 (52.6%)	3.1	(1.6)	4.0	(4.0)	97/181 (53.6%)	3.2	(1.6)	4.0	(4.0)	13/28 (46.4%)	3.1	(1.7)	3.0	(4.0)	150/443 (33.9%)	3.2	(1.6)	4.0	(4.0)	1.04 (0.93;1.16)	0.530	1.04 (0.89;1.22)	0.599
Volunteering	18/67 (26.9%)	2.4	(1.4)	2.0	(3.0)	51/177 (28.8%)	2.5	(1.5)	2.0	(3.0)	12/34 (16.4%)	2.8	(1.5)	3.0	(3.0)	81/278 (29.9%)	2.5	(1.4)	2.0	(3.0)	0.96 (0.83;1.10)	0.512	1.05 (0.89;1.24)	0.585
Unions	25/98 (25.5%)	2.3	(1.3)	2.0	(3.0)	67/247 (27.1%)	2.5	(1.3)	2.0	(3.0)	17/51 (33.3%)	2.3	(1.5)	1.0	(3.0)	109/396 (27.5%)	2.4	(1.3)	2.0	(3.0)	0.94 (0.83;1.07)	0.349	1.01 (0.87;1.17)	0.890
Experiences as patient	38/98 (38.8%)	2.7	(1.4)	3.0	(3.0)	38/204 (18.6%)	2.0	(1.2)	1.0	(2.0)	6/38 (15.8%)	1.7	(1.2)	1.0	(1.0)	82/340 (24.1%)	2.2	(1.3)	2.0	(2.0)	1.03 (0.90;1.18)	0.639	0.69 (0.54;0.87)	**0.002**
Information material	13/96 (13.5%)	2.0	(1.2)	2.0	(2.0)	52/242 (21.5%)	2.3	(1.3)	2.0	(2.0)	9/50 (18.0%)	1.9	(1.3)	1.0	(2.0)	74/388 (19.1%)	2.2	(1.2)	2.0	(2.0)	1.04 (0.91;1.17)	0.588	1.14 (0.98;1.31)	0.081
Experiences as relative	26/95 (27.4%)	2.4	(1.3)	2.0	(3.0)	33/213 (15.5%)	1.9	(1.2)	1.0	(2.0)	6/38 (15.8%)	1.7	(1.2)	1.0	(1.0)	65/346 (19.8%)	2.0	(1.2)	1.0	(2.0)	1.00 (0.86;1.16)	0.994	0.79 (0.63;1.00)	**0.047**
Media	12/97 (12.4%)	1.9	(1.2)	1.0	(2.0)	33/243 (13.6%)	1.9	(1.2)	1.0	(2.0)	7/53 (13.2%)	1.6	(1.1)	1.0	(1.0)	52/393 (13.2%)	1.9	(1.2)	1.0	(2.0)	1.01 (0.87;1.19)	0.866	1.00 (0.83;1.20)	0.958

^a^GP undecided vs. GP-non-committed, adjusted for gender and graduation university

^b^GP undecided vs. GP-committed, adjusted for gender and graduation university

## Discussion

### Summary of main findings

This study explores a range of factors associated with medical trainees being either positive or undecided about specialization in general practice during their transition from undergraduate training to basic clinical training. Outcome model 1 showed us that a future career that harmonizes with private life and provides the opportunity to be self-employed were significantly associated with participants being positive/undecided about a general practice career when compared to the GP-non-committed orientation. Further, autonomy over own working hours and a doctor-patient relationship based on continuity was associated with GP-positive/undecided orientation in outcome model 1 but favored GP-committed orientation in outcome model 2. In outcome model 2, however, the GP-positive/undecided participants were associated with an openness to alternative career paths in the future and positive attitudes towards using technology in their work and working when others are off duty. The participants’ specialty preferences, were highly influenced by experiences and knowledge about specialties obtained during medical school including the meeting with role models, and, for the GP-committed, also through their personal meeting with a general practitioner as patient or relative.

### Comparison to existing research

The GP-committed and GP-positive/undecided participants’ preferences for working hours and a doctor-patient relationship based on continuity corresponds both to the general descriptions and definitions of GPs’ work [6, 8, 30] and to factors that in former studies have been reported as reasons for choosing specialization in general practice [31–33]. Thus, our results indicate that medical trainees with general practice as specialty preference have an attitude to their future work life that aligns well with actual work in general practice [6]. Our findings also confirm the existing body of literature on the pivotal role that undergraduate exposure to the specialty plays in the general practice specialty choice process [16, 34–36].

Of particular note is that we find a relation between medical trainees’ specialty interest and an underlying wish to have autonomy over own working hours. Our data thus expands on findings in previous studies that report that the ability to determine own style of work and flexibility in work hours increases GPs’ satisfaction in their profession and serve as positive influences on career choice [30, 36]. According to the theoretical model by Bennett and Philips [13], the greatest recruitment potential lies within the GP-positive/undecided group. However, the literature review only briefly touches upon the potential influencing factors, and the recruitment potential is therefore not expanded in that study. We find that exposure to research activities had influenced the specialty preferences of more GP-positive/undecided participants compared to GP-committed which is consistent with findings in previous studies [37, 38]. One suggestion on how to benefit from the recruitment potential is, therefore, to increase focus on other dimensions of general practice as a specialty, such as academic teaching, research activities and the emerging use of technology like ultrasound [39]. This is likely to encourage some medical trainees to be recruited to general practice, while others not interested in research could be pushed away [37]. However, in the perspective of recruiting and retaining the physician workforce, such risk must be weighed against the view that ‘the biggest losses to general practice recruitment are those not ever considering it’ [40, 41].

In the local setting of this study, focus has also increased on the impact of a general practice-oriented undergraduate curriculum on recruitment of future GPs in Denmark the past years [31]. Further, interventions have been done towards increasing the extracurricular exposure to general practice by creating more jobs in general practice for medical students (students’ jobs) and junior doctors in Danish postgraduate training. This serves to promote exposure to general practice at all educational stages, and thus sharpen the pivotal elements of choosing the right career track [17, 19]. Nearly 30 years ago, studies reported that the desire to protect time for leisure and family, and concerns about an overwhelming workload were reasons for medical trainees not considering general practice [42]. Congruently with our findings, general practice trainees and recently qualified GPs in seven contries including Denmark report compatibility with family life and autonomy and independence as reasons why they in recent years have chosen general practice specialization [33]. This evolvement in the view of the general practice profession emphasizes a need for the surrounding environment to acknowledge that physician well-being and the work/life balance are important concerns today, also when it comes to choosing a specialty. In this way, young physicians tend to view the general practice profession differently than from an established specialist perspective [43]. This has been attributed to a generation gap [44], where young physicians’ desire for a good work/life balance conflicts with the demands of accessibility and continuity in general practice [30].

In the present study, we identify associations between interest in a general practice career and importance of the opportunity to be self-employed even though it is only rated important by less than half the GP-committed participants. This indicates that self-employment is not a cardinal issue during undergraduate education, and that autonomy and the opportunity to form own work life might be more important to them at this time than having the opportunity to be self-employed in the future.

### Strengths and limitations

The present study is strengthened by its methodological and theoretical grounding of a systematically developed and content-validated instrument with main outcomes being guided by a theoretical framework [45]. Furthermore, the study was conducted on a national cohort of medical trainees educated from all four medical schools in Denmark. However, the study is limited by its quantitative nature and cross-sectional design. Thus, we can only report intentions about specialization and attitudes towards future work life at a stage of medical education where the participants have no or limited working experiences. Furthermore, the response rate just below 40% implies a risk of selection bias, however, the non-response analyses previously reported found the study population to be representative of the total population of medical trainees beginning basic clinical training in Denmark [22].

### Implications for further research

Further research is needed on the development of specialty orientations and hence intentions to become a GP over time. Such longitudinal examinations would contribute to knowledge about the stability of the specialty orientations and associated factors over time as well as the dynamics of influencing factors after the transition into postgraduate medical training. Further, we recommend future qualitative studies to explore how the perceived work fits the experienced work in general practice and to deepen the understanding of the perceived barriers to become a GP.

## Conclusion

This study set out to explore the recruitment potential of medical trainees who are positive towards or undecided about specialization in general practice. We found that this group of participants value work/life balance and autonomy over own working hours more than the GP-non-committed participants, but less than the GP-committed trainees. The GP-positive/undecided orientation, however, is associated with a positive attitude towards technology, working shifting hours, and an openness towards several career paths when compared to the GP-committed orientation. Furthermore, participants’ specialty preferences are highly influenced by undergraduate experience. Our results, therefore, indicate that recruitment to general practice specialist training could be increased by prioritizing such undergraduate experiences in general practice along with a rise of attention to the diversity of career opportunities that are open to a specialist in general practice.

## Data Availability

The datasets used and/or analyzed during the current study are available from the corresponding author upon reasonable request.

## Abbreviations

GP: General practitioner
GP-committed: Medical trainees with general practice as a preference for specialization
GP-non-committed: Medical trainees who have excluded general practice from specialty considerations
GP-positive/undecided: Medical trainees who are positive towards or undecided about general practice specialization
